# 4D-BIM-Based Workspace Planning for Temporary Safety Facilities in Construction SMEs

**DOI:** 10.3390/ijerph17103403

**Published:** 2020-05-13

**Authors:** Kieu-Trang Pham, Duc-Nghia Vu, Phuc Le Hieu Hong, Chansik Park

**Affiliations:** 1 School of Architecture and Building Science, Chung-Ang University, Seoul 06974, Korea; kieutrangnuce@gmail.com; 2 School of Computer Science and Engineering, Chung-Ang University, Seoul 06974, Korea; dnvu@uclab.re.kr; 3 Autodesk Asia Pte. Ltd., Singapore 138633, Singapore; phuc.le@autodesk.com

**Keywords:** temporary safety facilities (TSFs), safety planning, workspace planning, safety management, 4D-BIM

## Abstract

Temporary safety facilities (TSFs) are an essential support system providing necessary protection to workers during construction activities, which are targeted towards preventing the occurrence of incidents and accidents at the construction site; however, the schedule and location of installation and demolition of TSFs continue to rely on labor experience, and are often omitted from formal drawings or documents. This results in thousands of accidents in the construction industry, especially in construction small and medium-sized enterprises (SMEs) because of their several limiting factors; therefore, this study proposes automatic workspace planning for TSFs based on construction activities, which is a systematized approach for construction SMEs to practice occupational health and safety (OHS). By using building information modeling (BIM) and add-in algorithm, safety facilities can be simulated and visualized to integrate into the designated workspace. The developed system was implemented utilizing 4D-BIM for TSFs installation and validated with a case study on a residential building project. The result revealed that the visualized TSF produces a better understanding of safety measures with regard to project schedule. Additionally, TSFs workspace planning provides an affordable approach that motivates safety practices among the SMEs; consequently, the effectiveness of construction safety measures and their management is enhanced appreciably.

## 1. Introduction

**Figure 1:**
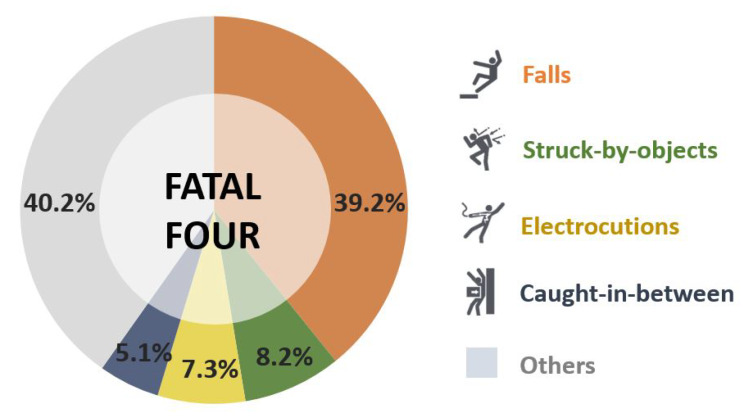
Proportion of accidents attributed to Fatal Four in construction, as reported by the Occupational Safety and Health Administration (OSHA) 2018.

The integration of innovative technologies in the field of information and communication technology (ICT) is rapidly transforming conventional construction into smart construction. Nonetheless, the nature of the construction process makes it prone to risks and accidents; moreover, innovations in the construction sector have given rise to new safety issues. According to recent public reports, construction is one of the most hazardous occupations with considerably higher number of work-related injuries and fatal accidents than other industries [1,2,3]. The Occupational Safety and Health Administration (OSHA) reports show that worker fatalities in the construction industry in 2017 accounted for 20.7% with 971 cases. Indeed, among the hazards that account for injury and death, OSHA has labeled “Fatal Four” as the leading causes of workplace deaths, which are responsible for more than 60% among the construction worker deaths, by the US Bureau of Labor Statistics (BLS) reports. These four main hazards consist of falls, struck-by-object, electrocutions, and caught-in-between [4]. Figure 1 shows the proportion of accidents attributed to each category in the Fatal Four.

On the other hand, the planning of occupational safety is determined by the size of the construction site, system of work, and project delivery methods. A comparison among firms of various sizes shows that a high proportion of accidents in terms of number or severity occurred in small firms [5]. The literature review revealed that there is no standard definition for determining a construction small and medium-sized enterprises (SMEs), there are differences among countries and industries [6]; however, SMEs can be defined by some common criteria that comprise the number of full-time employees and annual sales are below a certain level. For example, in the European Union (EU) a company with fewer than 250 employees is considered an SME, while in the United States (USA) an SME has fewer than 500 employees. According to the Annual Report on European SME in 2016 and the USA Small Business Administration Statistic in 2015, the proportion of employment in construction SMEs made up for 87.6% and 82.7% respectively [7,8]. It means that the improvement of safety management in construction, SMEs play a critical role to safeguard more than 80 percent of workers in the construction industry. There are various factors influence the safety performance in construction SMEs, including the number of laborers, limited availability of skilled labor, less years of experience, resource availability, poor safety attitudes, size of the project, contract value, and time and budget for construction [9,10]. Accordingly, this imposes several constraints that lower the commitment of SMEs to implement safety programs. Thus, the actual health and safety practices in the construction sector need to be investigated from many aspects [11]. Normally, SMEs are involved in all sequences of the construction process. The shortage of technically skilled and experienced laborers makes the SMEs more prone to adopt adverse safety practices [9]. Regardless of the many guidelines provided by OSHA and resources available for improving safety management, the compliance of these safety regulations in practice remains reactive rather than proactive. Owing to the overwhelming number of safety rules, identifying the appropriate safety guideline and delivering them to the right personnel or right working place becomes a challenge. Considering the abundance of safety guidelines in construction, workers or even inexperienced managers may be unaware of or ignore some safety requirements during construction operation [12]. Furthermore, it is undeniable that safety awareness is not sufficiently high in construction SMEs. Consequently, issues such as forgetting to adhere to safety guidelines and misunderstanding the guidelines are common, resulting in accidents. Besides, designing safety planning requires the involvement of safety professionals; however, SMEs claim that they do not have the necessary funds to engage specialist human resources. To overcome this, SMEs need to follow a strategic method to drive the adoption of new technologies and building information modeling (BIM) processes.

On the other hand, these traits are not all negative, they also have positive points. SMEs could have more potential than larger companies in simplifying the incorporation of BIM by converting their drawbacks into opportunities [13]. Although SMEs have limited human resources, they attract new and young workers into the sector, who are interested in novel technologies and innovative ways of working. With small to medium amounts of construction information, SMEs can be more agile and simple than larger firms in implementing, syncing, and integrating digital information. Additionally, decisions are generally made more quickly and more manageable in small-scale organization rather than an extensive implementation with numerous reviewed time and disruption. In fact that a lot of construction SMEs, especially start-up firms have been proactive catch up with BIM technology and new tendencies, enhancing their competitive ability and narrowing the gaps with larger firms. As a result, the application of BIM in SMEs is an inevitable trend for the future construction industry.

This paper proposes a 4D-BIM-based automatic workspace planning which allocates temporary safety facilities (TSFs) for designated model objects/conditions integrating activity-based hazard evaluation and its corresponding prevention. The assessment data were arranged specifically by the severity rate of accidents related to individual activities and then the correlated protection was calculated. The proposed approach was applied to a residential building project. It was found that 4D-BIM software platforms could automatically place the TSFs at the right time and right place. It is expected that the system can provide general safety planning without the participation of safety experts. Consequently, it decreases the manager workload, improves safety management, saves SME resources, and helps construction SMEs to adopt a closer approach to a smart and safe working environment.

The remaining paper is structured as follows: Section 2 poses the state-of-the-art studies performed in this research domain. The section discusses the importance of construction fatality data analysis for safety planning, application of TSF in construction SMEs, BIM-based safety planning, and need for an effective approach for safety planning in construction SMEs. Next, the proposed framework is presented in Section 3. Section 4 discusses the data assessment process. Then, Section 5 gives the research design for the proposed system. After that, a case study of a residential building project is presented in Section 6. Section 7 discusses the proposed system evaluation results. Finally, the limitations of this study along with the conclusions and direction of future research are presented in Section 8 and Section 9 respectively.

## 2. Literature Review

The section below sets the scene by giving an overview of the existing research on accident data analysis, temporary safety facility, and initiatives concerning BIM-based safety planning in the construction industry.

### 2.1. Importance of Causality Analysis of Construction Fatality

Certain research has underlined the importance of identifying the cause of accidents and determining their role to prevent accidents from happening. Betsis [14] highlighted correlations among various accident characteristics with causes and construction stages. Recent studies showed that fall-related hazards were major concerns [15,16,17], and most construction accidents and fatalities were in relation to small or medium firms [14]. Arboleda and Abraham [11] analyzed the causes of accidents to find the major relationships between the types of accident models and behavioral causes resulting in trenching fatalities. Many studies have focused on analyzing hazards based on the severity of incidents, their frequency, and risk distribution [16,18]; however, the investigations provided only the relevant information without discussing a practical application for safety planning purposes. For example, Memarian and Mitropoulos [19] pinpointed the contribution of masonry activities to accidents and identified high-risk activities. The results indicated that the majority of incidents in masonry work occur during block laying; the study also provided recommendations for reducing such incidents. Nevertheless, the practical applicability of these results to safety planning is not discussed. Besides, the practical approaches that are currently available do not focus on a method to integrate accident data into the BIM by schedule timeline. Kim et al. [20] analyzed work-space conditions associated with safety hazards of scaffolding, which are automatically shared in BIM. In this study, a proposed framework was specialized in scaffolding work, it was combined with a safety checking program. Another research by Kim et al. [21] automated decision making for choosing appropriate temporary structures designated for scaffolding work; however, the study did not include sufficient safety planning that enables the use of various safety facilities in the entire construction process. Hence, efforts are needed to investigate the feasibility of integrating safety measures by incorporating advanced technologies into adequate safety planning processes, which adopt a simple approach to encourage widespread use (easy to use, update, and modify) and allow the automatic selection of temporary safety measures for a specific working condition.

### 2.2. TSFs in Construction SMEs

TSFs are essential equipment/facilities used for a limited period, depending on the change in constructing conditions, for the purpose of providing adequate safety and prevention at a construction site. Although extensive research is available on the planning of permanent structures in construction, temporary structures continue to be overlooked or receive minimal consideration [22]. Hence, there remain problems that need to be solved by an in-depth investigation using potential technologies. Frequently, hazards arise from the operation of temporary structures, partly because of the subjective thinking that activities related to these structures are secondary tasks [23]. Most previous studies reveal that temporary structures were installed without adequate planning [20] because the existing safety planning neglected to consider safety problems in using temporary facilities. Devising a paper-based plan for a temporary safety structure is definitely a time-consuming, ineffective, and labor-intensive process. This work requires the preparation of various documents for detailed planning with reviews of several aspects such as building geometry, design generation, type of structure, and impact on time and cost [21]. Considering the shortage of time, cost, and labor in construction SMEs, the execution of safety measures need to be a concern in these enterprises.

On the contrary, by comparing the distinct characteristics of the firm size, particularly with regard to safety management and execution, several studies have emphasized that weak occupational health and safety (OHS) practices resulting in high risk of accidents at construction sites were commonly observed in SMEs. Some papers claimed that the biggest restricting factor in construction SMEs was skilled labor, whereas others attributed this to cost [9,17]. Additionally, the legal regulation for construction safety focuses mainly on large enterprises, and managerial responsibilities are often simplified for SMEs. Although several papers have considered the barriers to safety implementation in SMEs [24], none of them have provided practical solutions for such problems. Besides, temporary facilities of different price ranges and intended for different purposes are commonly used in construction sites of various sizes. A construction company chooses a suitable subcontractor or supplier for their construction based on their financial condition; therefore, the type of these facilities in each company may be different and safety planning should consider the dynamic update of the TSF family library on their server. Nonetheless, no comparable studies exist for automatically allocating TSFs in workspace planning in small constructions.

### 2.3. Bim-Based Safety Planning

Emerging as a promising visualization technology, the BIM application has paved the way for safety performance by its capability to deliver a rich profusion of digital construction data [25,26,27,28]. The innovation of 4D-BIM is recognized as an intelligent collaboration link between the 3D digital model and schedule information [29,30]. This environment accommodates the visual information containing highly collaborative parameters that potentially simulate safety features [31,32]. Embracing BIM technology gives SMEs the opportunities to drive down both direct and indirect costs and deliver competitive quality outcomes. Most existing studies on BIM and safety planning cover a wide range of safety applications in construction. Several researchers have explored ways to facilitate smart and safe execution throughout the construction process [33,34,35]. Additionally, the continuous improvement in the BIM technology has supported collaboration and made sharing of data faster, easier, and more efficient [34,36,37]. Table 1 summarizes the state-of-the-art applications of BIM technology for construction safety planning. The studies demonstrated effectiveness in using BIM for automatic hazard analysis. Kim et al. [20,21] focused on scaffolding hazards, whereas Wang et al. [38] concentrated on excavation and Zhang et al. [39,40] focused on fall hazard protection. The integration of BIM and rule-based analyses from subjective regulations preferably apply to medium or large construction enterprises for specific and professional safety planning. In contrast, SMEs require a simple and familiar approach that effortlessly adapts to their operation process. Thus, automatic workspace planning of safety facilities using BIM for construction SMEs is a new topic that has not been investigated.

### 2.4. Need for an Effective Workspace Planning for TSFs Based on 4D BIM Platform

With construction rapidly becoming a data-driven industry, digitizing construction information is one of the first steps toward smart management. Accordingly, to enhance the competitive ability in the construction market, SMEs have to transform their conventional method into smart construction. Nevertheless, most of construction SMEs operate under several constraints such as time, cost, and the shortage of qualified personnel, which restricted them to utilize expensive innovative technologies in construction operation [41], particularly for safety applications. Besides, considering the variety of safety information, selecting an appropriate temporary facility to prevent hazards related to each specific activity requires contractors to have experienced and employed skilled laborers. Thus, to facilitate smartly implementing safety requirements in construction SMEs, it is essential to have a method that effortlessly determines when, where, and what kind of safety facility needing to install or remove in a specific workplace. The integration of BIM in safety planning process brings potentials that aid SMEs to not only increase their productivity, labor-saving, standardize the data scheme, and deliver it consistently when involving in repeat projects but also improve their reputation and operation quality to compete with bigger firms. It is expected that the system with BIM integration is an affordable approach for SMEs because its long-term benefits and advantages bring to SMEs is far ahead than invested cost. From this motivation, our study proposes an approach to automatically conduct activity-based workspace hazard identification and then navigate related TSFs by employing a familiar BIM technology. To accomplish this, the study creates a plug-in compatible with Revit and Navisworks, which are familiar platforms currently used to design and export technical drawings in almost all construction firms. With the effective association of a graphical algorithm in the BIM platform, the TSFs relating to distinct conditions can be automatically simulated in a 4D-BIM environment in real-time.

## 3. A Comprehensive Framework for Proposed System

**Figure 2:**
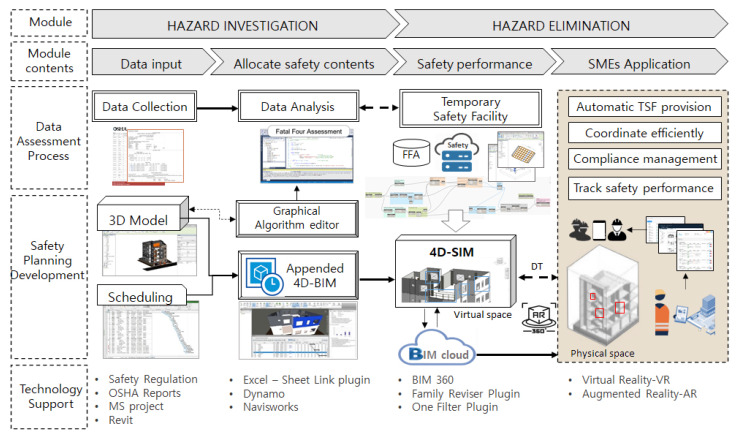
Proposed framework for workspace planning for temporary safety facilities (TSFs) (WPT) system.

To address the aforementioned problems, this study aimed at developing and validating a concept prototype of 4D-BIM-based automated workspace planning for TSFs (WPT) system by considering the severity of activity-based hazard and its corresponding prevention with regard to specific spatial and temporal conditions. Figure 2 illustrates the comprehensive framework of developing WPT system, which can be understood in both horizontal and vertical directions. The vertical direction presents each steps in workflow of the system while the horizontal direction show how to implement these steps. The vertical direction including data assessment process and workspace planning process, are presented in Section 4 and Section 5 respectively. The main contribution of the former process was to identify proper activity-based TSFs, then the later process takes advantage of BIM and the former’s results to develop safety planning in the virtual space. The horizontal direction adopted two main modules of hazard investigation and hazard elimination are explained as follows:

- Hazard investigation module. This module consists of the following two-stage process: data input process and allocating safety contents process. The input data process is a preparation of project 3D model and 2D data such as project schedule and the Fatal Four data collected from the OSHA website. Following this, the allocating safety contents is a procedure of analyzed and assessed Fatal Four data. Concurrently, the designated tools for 2D and 3D data exchange such as a graphical algorithm and BIM were employed for developing the linking of databases for the next module.
- Hazard Elimination module. The safety performance process was developed by 4D-based safety information modeling (4D-SIM). Relevant safety contents consist of safety regulations and prioritized TSFs are displayed in the visual environment, in which serious hazard notifications and model-linked TSFs automatically shown by construction sequences. The accessible approach was adopted by uploading 4D-SIM data on the company’s server using the BIM cloud. Finally, it can be seen from the beige box in the last process, 4D-SIM data can be integrated with augmented reality (AR) for the purposes of safety monitoring at construction site. In this practical application step, the required TSFs for specific activities were uploaded and inquired from BIM cloud server. Then, the users can approach the WPT application everywhere by accessible devices such as a tablet, mobile phone, monitor, etc. The AR can connect to these devices for supervision purposes. The integration of BIM and AR creates a visual interaction for users to read and update safety facilities information to facilitate AR part for monitoring the installation and removal of TSFs in real site construction. More details of this collaboration process will be developed and clarified in further study.

## 4. Data Assessment Process

**Figure 3:**
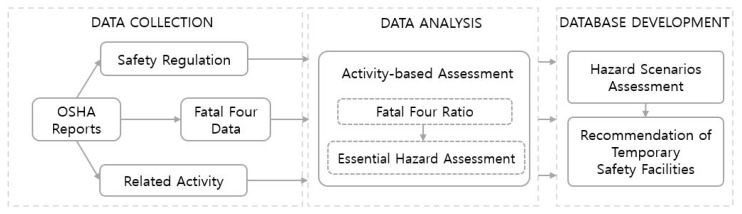
Fatal Four assessment (FFA) process.

Assessment systems include several steps, which aim to indicate the severity of hazards in each work activity. Then, the highest hazard is addressed along with proper prevention method. Figure 3 explains the detailed steps to perform the Fatal Four assessment (FFA) process.

### 4.1. Fatality Data Collection

To develop an assessment database, this study considers a broad expanse of reports on construction accidents to identify the essential hazards in each working activity. Various methods are available for collecting and analyzing the occurrence of accidents, which aim to clarify the cause of an incident based on subjective aspects (such as organizational level, control measures, installation and maintenance, training, and management factors) or objective problems such as environmental issues [42,43]. Fatality reports are available from various sources such as OSHA, Bureau of Labor and Statistics (BLS), and the National Institute for Occupational Safety and Health (NIOSH). NIOSH reports depend on death certificate data, whereas OSHA and BLS gather information related to investigations of all work-related fatalities from employers or the news media. This study chose a database available in the OSHA website, which provided detailed data with characteristics and variables of fatality cases [44]. The input data were retrieved as is from the database included the name of the report, type of accident, time and location, age and employment status of the victim, geographical region, size of job-site, nature of the project, reason for the incident, and recommendation (as illustrated in Table 2).

### 4.2. Theoretical Analysis of Fatal-Four-Related Hazards

Hazard identification and assessment play a crucial role in the OHS program. Failure to recognize hazards is one of the root causes of injuries, accidents, and incidents in the construction site. Therefore, this data analysis step aimed at indicating the main potential hazards in work sequences to prevent future occurrences of accidents. By thoroughly investigating the accidents and fatality reports, we can assess and determine essential hazards that might potentially happens for each activity. The activity-based assessment was conducted by evaluating the severity of the potential hazard outcome and prioritizing the hazards. This process includes two steps:

1. Fatal Four Ratio: From OSHA data reports, the percentage of fatality cases corresponding to each Fatal-four type (i.e., falls, struck by, electrocutions, and caught-in between) is determined for each construction activity.
2. Essential Hazards Assessment: The essential hazard is a type of Fatal Four that has high possibility to happen. It is determined by Fatal Four type that has highest percentage from the Fatal Four ratio.

In the database development step, a database that includes the recommended TSFs is developed corresponding to hazard scenarios assessment. After obtaining the essential hazards for each activity from previous step, related TSFs is recommended based on the hazard scenarios according to OSHA regulations [45]. The criteria to classify what kind of TSFs in relation to Fatal-Four-related hazards were presented in Table 3. For an example, in roof coatings activity, after analyzing the OSHA reports data, it is observed that Falls is the Fatal Four type that has highest percentage of occurrence. Hence, it is an essential hazard. If it is happen in wall openings scenarios, the guard railing is recommended for safety protection.

### 4.3. Database Assessment System Implementation

The scenario assessment was conducted by evaluating 430 construction fatality cases which were attributed to the Fatal Four among 1266 fatality cases reporting by OSHA in 2018 [44]. By classifying activities based on the Fatal Four, accident rates and the main causes of fatalities in a specific work activity were identified, and then best practices for prevention of such accidents were recommended. The collected Fatal Four data were investigated to assess the possibility of occurrence of major accidents according to the project schedule. The data were presented according to the severity and frequency to assess the possibility of occurrence of the main accident in each stage of the project schedule.

**Figure 4:**
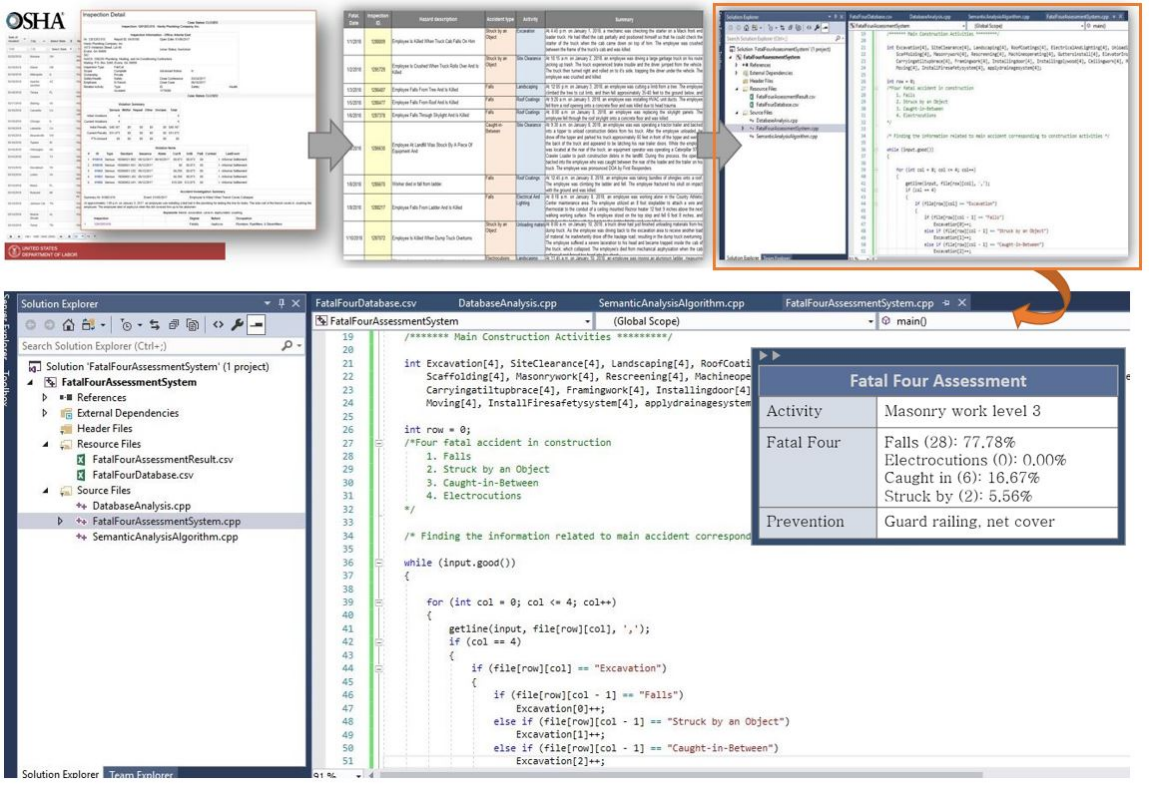
Fatal Four assessment process.

An FFA system was developed to analyze the Fatal Four data and output the database of the recommended TSFs related to activities with a high rate of accident occurrence. From input data of the Fatal Four collected from OSHA reports, FFA system uses semantic and textual algorithms to analyze and find information related to accidents for each activity (Figure 4). Next, the FFA system computed the possibility of occurrence of each type of accident based on the obtained number of accidents for the activities from the data analysis. After that, essential hazard is determined. The FFA system was implemented using the Visual Studio programming tool. TSFs are recommended to prevent frequently occurring accidents. The information from database is extracted and integrated in 4D-BIM safety planning. This step is explained in detail in the next sections.

## 5. Design for WPT System

### 5.1. WPT System Development

The workspace planning for TSFs (WPT) system was designed to introduce activity-based safety facilities that appear in a specific condition during the construction process. The developed WPT system utilized five software applications: (1) Autodesk Revit 2018: a BIM tool to stored, visualized, and managed digital information related to building and construction process; (2) Dynamo 2.0.2: a Visual Programming Language (VPL) that extents the power of Autodesk Revit; (3) Microsoft (MS) Excel 2016; (4) MS Project: a project management software product; (5) Navisworks Manage 2019: a BIM coordinator solutions.

**Figure 5:**
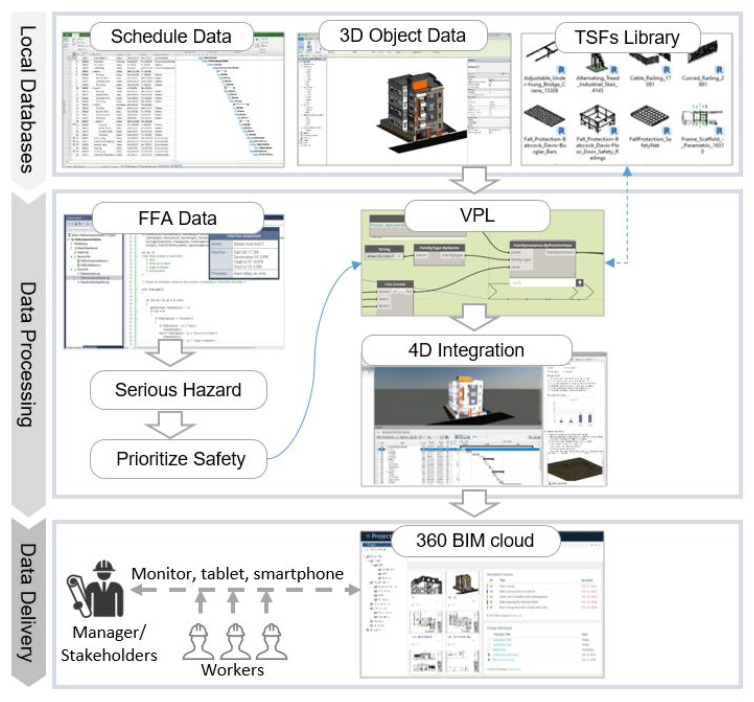
System architecture.

The process of data procedure in the WPT system includes three stages: local database preparation, data processing, and data delivery to users. The WPT system architecture is presented in Figure 5. Each company has their own local databases, which are obtained from specific project information and TSFs library, which is variously provided by the supplier on the construction market. The data processing stage utilize FFA data to identify a serious hazard and select prioritize safety prevention in construction sequences. Regarding the BIM attribute addition, Dynamo can recognize and specify the time for invoking the model components. Accordingly, the coordinated 3D-BIM building model and TSFs library were extracted in real-time. This function enables access to the Revit .NET API application programming interface, which allows programming with any .NET compliant language including VB.NET, C#, and C++/CLI. It permits the user to query data, change element properties, and add/modify certain components directly from the visual environment. The last stage allows updating 4D-BIM-based data from the system to the company network through the cloud server by using the 360-BIM cloud. This platform allows users access readily to WPT system from anywhere by personal devices such as tablets or mobile phones.

### 5.2. Linking Process of Contents in WPT System

**Figure 6:**
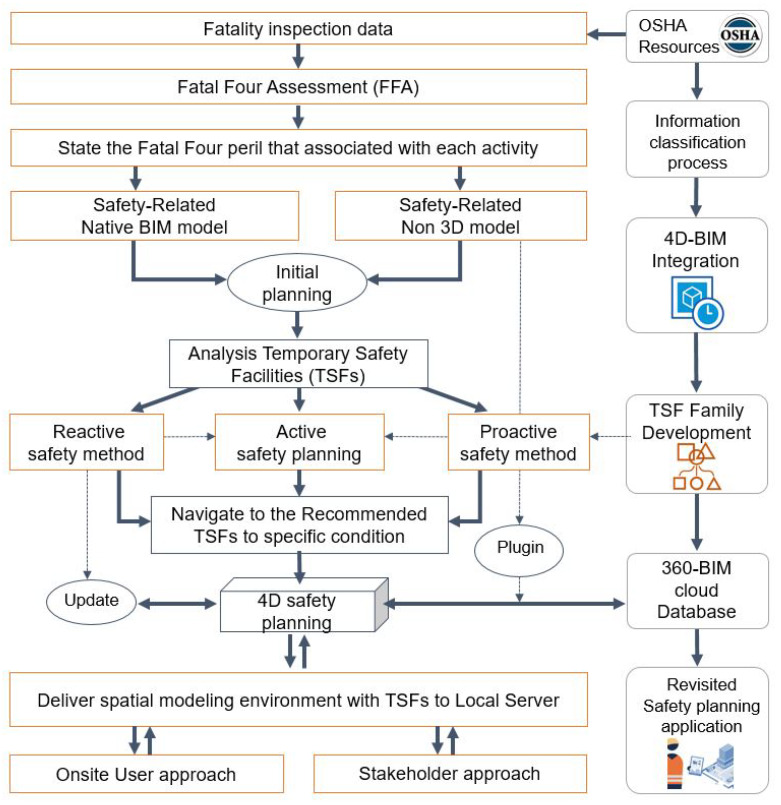
Information flowchart of the process of generating TSFs.

This section focuses on the details process of the development of data exchange procedures which coordinated the allocation of selected TSFs in WPT system (Figure 6). The integration of an automation tool with pre-written scripts into the BIM environment of VPL assists in conducting digital information management effectively, especially safety visual information. The VPL uses a block-based program and is designed with predefined functions related to model components in a BIM environment [33,46,47]; therefore, this paper employs Autodesk Dynamo which enables both textual and visual programming, and allows users to simultaneously code and design 3D models [48]. This user-friendly data interface programming enables to establish bilateral integration between Revit and MS Excel. Additionally, it distinguishes between blocks based on their shapes and colors and uses wire connections for inputting and outputting data.

### 5.3. Logic of TSF System Development

**Figure 7:**
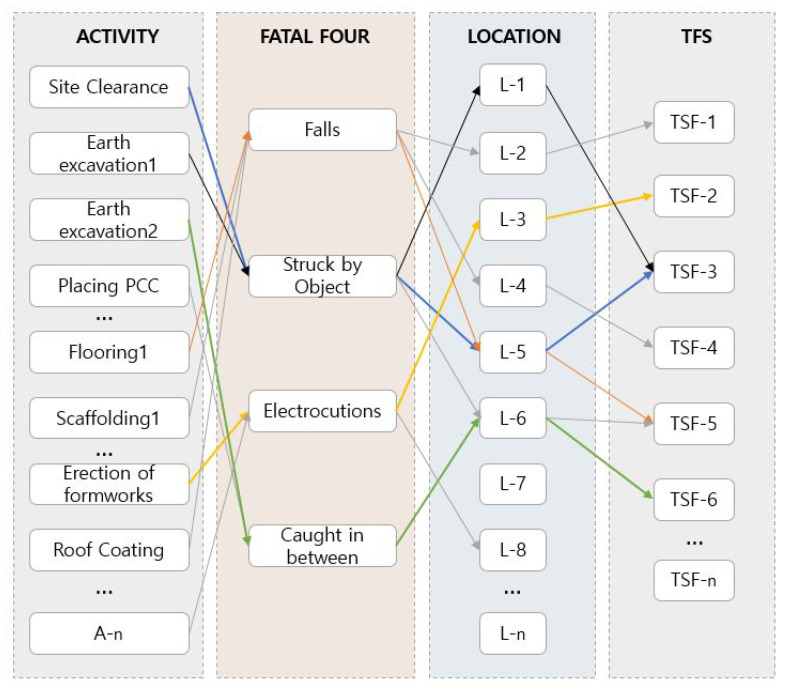
Logic of TSF system development.

In order to guarantee workplace safety, the participants, including managers and workers, have the responsibility to understand and meet several safety requirements. Navigating the TSFs system to specific work activity in a 4D-SIM framework depends on the relationships between three factors, i.e., the relationship between activity and FFA result, and that between the FFA result and subjects (location and space). The available TSFs that satisfy both these two linking factors are recommended for accident prevention. Figure 7 illustrates the relationship of activity-based FFA and TSFs recommendation. Consequences, the provision of both the schedule-based model and the activity-based essential hazard producing the prioritized safety protections in the work sequence. The interconnected workflows within these relationships were essential for developing the workspace planning for TSFs.

## 6. Case Study for Automatic Installing TSF System in a Residential Building

### 6.1. Case Scenario

**Figure 8:**
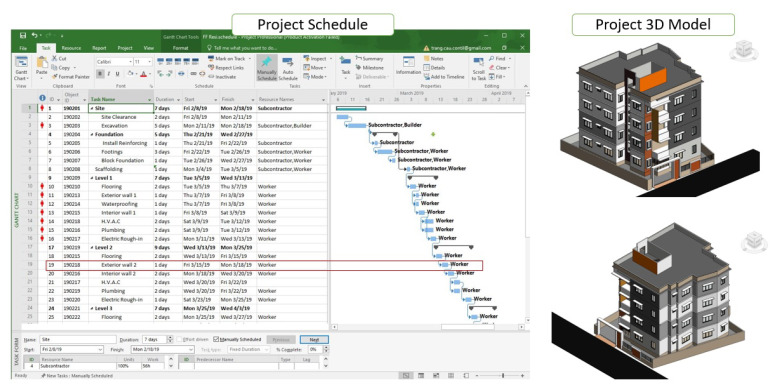
Residential building schedule.

The field safety case study was performed during the construction of a private residential building project with a total area of 320 sq. m and construction density of 60 percent. The example given in this case study is BIM model of a five-story building developed in Autodesk Revit software. It is a complete model with architectural and structural elements (without mechanical, electrical, and plumbing (MEP) equipment and furniture), and the project schedule was planned for six months (as shown in Figure 8). The construction of the exterior wall involves several activities including layout, material handling, scaffolding, block laying, block cutting, grinding, shoring, and grouting [19].

**Figure 9:**
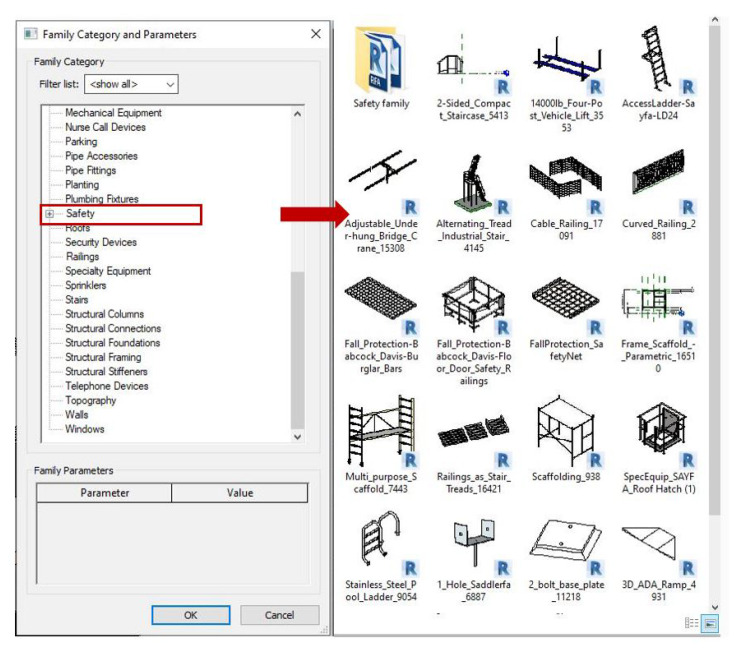
TSF family library.

The activity considered in the case study was block laying and the location was the floor slab edge. FFA results indicated that “Falls” were the main fatal cause, accounting for 77.78 percent of the accidents (refer Figure 4), and the hazard cause in this situation was unprotected sides. After identifying the main causes of fatal accidents during the construction of an exterior wall, measures to protect against falls were considered by following regulation OSHA1926.500 for the dimensions and application guidelines of the guardrail system (Table 4). It is assumed that the TSF used in this situation is a guarding system available in the local family library (refer to Figure 9). It is also assumed that the work for the main structural elements such as the structural floor, structural column, and beam system are complete. In the subsequent constructing stage, the exterior wall will be built at each floor elevation by day. To ensure the safety of laborers at the construction site, the system will be required to install a temporary railing system on the outer edge of each floor.

### 6.2. Automated Linking of the Excel Data to the BIM

**Figure 10:**
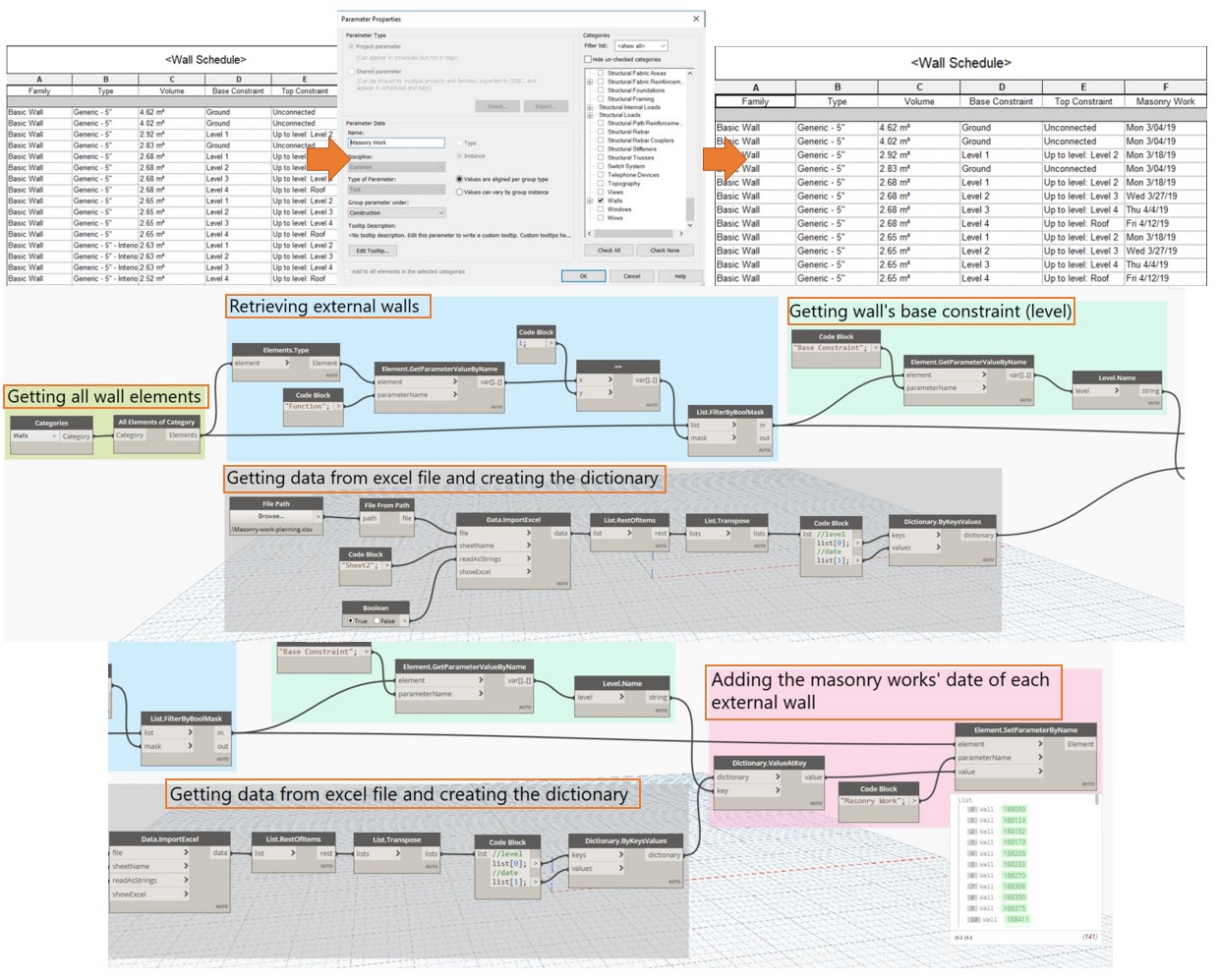
Wall schedule integration.

In the Revit model, the first step was obtaining the data (masonry work planning) from the project schedule that was made in Excel format (.xls). Accordingly, this information was added to all external walls through visual scripting using Dynamo. The categorical process of schedule integration with the exterior wall as illustrated in Figure 10. The parameter named “Masonry Work” is the project parameter in Revit, which is an instance and no data exist before running the first Dynamo script. The first script performs the following tasks:

- Retrieving all wall elements including external and internal walls in the Revit model.
- Retrieving all external walls. In Dynamo and Revit API term, each type of wall has a different function: the external wall function is 1 and the internal wall function is 0. Based on this, the “List.FilterByBoolMask” node was used to filter and capture all external walls in the model.
- For each external wall, the next node obtains the constraint-based of walls, which is wall elevations.
- At the same time, Dynamo processes all data from the Excel file consist of the planned date of constructing each wall elevation and creates the “dictionary” in the visual program environment. In this case, a dictionary is a data type composed of a collection of the key-value pairs, instead of an index value like in a list. Dictionary has no ordering of data and the user can look up data using a key instead of an index value, as in a list. In particular, the dictionary has elevation as the “Key” and the corresponding date for constructing the external walls at this elevation is the “Value”.
- Accordingly, Dynamo will obtain the Value (the corresponding date for building each wall) at the specified Key (the wall elevation). The “Element.SetParameterByName” will automatically add the information (masonry work date) to all external walls.

### 6.3. Simulation of TSF System with Arbitrary Constructing Time

**Figure 11:**
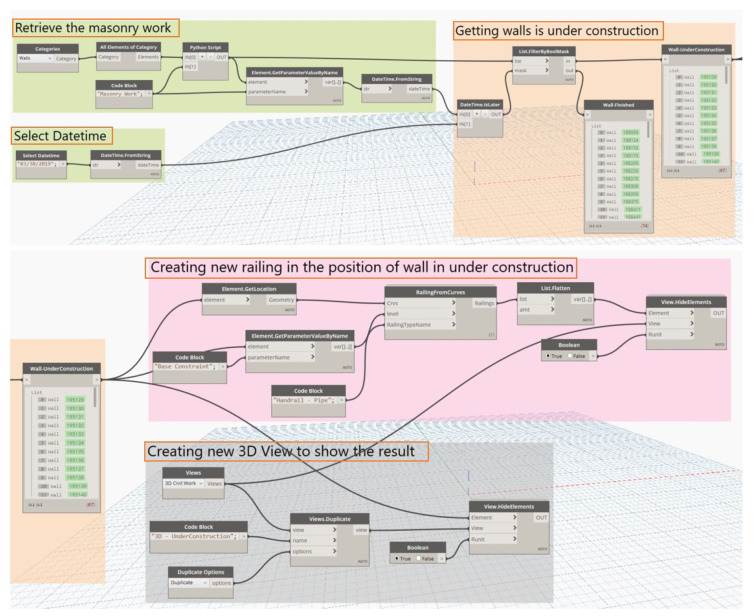
Placing temporary railing algorithm.

According to OSHA regulations (see Table 4), if the work level on the ground corresponds to the work level of 0 m or lower than 2 m, and the height of the wall from the floor level to the surface of the wall exceeds 2.4 m, then the limited zone is needed but a guardrail system is not needed. In addition, the following cases are considered: case 1: if the working level is higher than 2 m (case 1), and the wall has not been constructed, then the guardrail system and limited zone are needed; case 2: if the wall height exceeds 2.4 m and it is a solid wall, then only zone limitation is needed; case 3: if the wall height exceeds 2.4 m and the wall has an opening, then both limited zone and guardrails are needed. Hence, following the defined cases which were extracted from the OSHA regulations, the developed visual algorithm automatically allocate safety prevention at the distance higher than 2.4 m and visualize the case scenario along with the placement of the TSF system (shown in Figure 11).

The Dynamo script will depend on the progress of the schedule to automatically calculate and propose safety measures. A safety system that employs 3D BIM related to the activity of block laying was developed. The system allows synchronization of the model data back and forth between Autodesk Revit and the schedule spreadsheets. In this process, the schedule, activity, and the corresponding safety information ID are set up and loaded to the system database. The database contains Fatal-four-related fatality cases, the activity-based essential hazard, hazard scenario, and corresponding safety measures. Combining with the developed parameter model in Revit, the design team employs Dynamo to read available schedule data of exterior walls from MS Excel format. This VPL will automatically retrieve and integrate data to acquire information about the by-floor construction progress of architectural walls (includes exterior and interior walls, excludes structural walls and other walls). The user can choose any work dates in the schedule to estimate the needed tasks and continue using Dynamo to distinguish between the constructed walls and those under construction.

**Figure 12:**
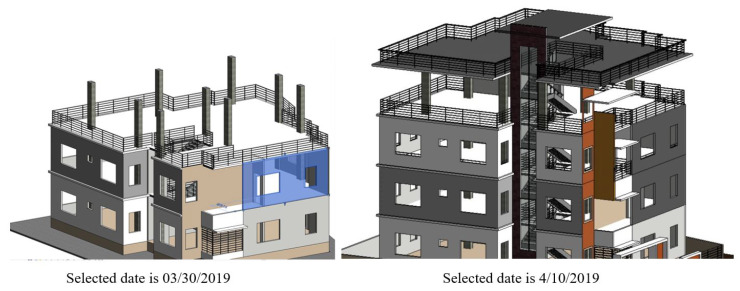
Automatic placing TSF by date.

After retrieving all walls that are not yet constructed, a new railing system is constructed at the positions of these walls. The new 3D view was also created to show the TSF system in the Revit model. Figure 12 illustrates the comparison of the corresponded temporary railing system on the selected dates of 30 March and 10 April. According to the schedule, the external walls at floor elevation 3, 4, and the roof have not been constructed; therefore, temporary railing systems are installed at these elevation levels. If the selected date is changed, Dynamo will automatically create a new 3D view in Revit to simulate the temporary safety systems at that time.

**Figure 13:**
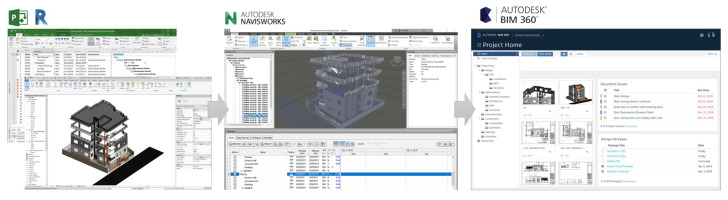
Process of delivering TSF on local network.

As illustrated in Figure 13 formerly prepared TSF workspace planning was simulated on the loaded data from the Revit into 4D-BIM environment in Naviswork for real-time simulation purposes. Then, for the purpose of improving interaction with users, data was up to date on cloud servers by 360-BIM platform. Finally, with the BIM and Computational tool, Project managers and safety managers will quickly visualize and manage information as well as the schedule of installing safety equipment at the construction site.

## 7. WPT System Evaluation

Following the prototype development of the WPT system, an experiment was conducted to validate the usability of the WPT system. The process commenced with small training, whereby the WPT system functions and features were explained, and a workspace planning for the residential project was delivered, giving participants an overview of this visual safety planning to acquire safety information pertaining specifically to required TSFs in the construction process. After that, the participant took part in the survey through questionnaires and interviews related to the criteria for the usability of the WPT system. These criteria were given to evaluate the characteristics of WPT system that are particularly useful for SMEs’ access. A group of 18 participants including contractors of construction SMEs (three companies in Korea and seven companies in Vietnam), three Vietnamese site supervisors, and five construction managers from Iraq, Pakistan, and Vietnam were asked to experience the WPT trial platform. Trials were conducted with two methods, utilizing the conventional paper-based approach and the WPT system approach, with survey questionnaires. Subsequently, through issued questionnaires, interviewees evaluated the potential of the WPT in terms of the following seven criteria [26,28,49,50]:

1. Understand: How well do the users understand the system? Considering the participant’s ability to understand safety requirements and its implementation through these approaches.
2. Feasibility: Is this system capable to be implemented in your company? Representing the usability and applicability of the proposed system in construction SMEs.
3. Cost: Do you think this system is costly? Considering the long-term impact on the cost of using this system including labor-cost, rework-cost, and other indirect costs.
4. Ease: Is this system easy to use? Considering participants’ senses when using the interactive visualization system and paper-based system. The safety information can be approached easily by flexible access from personal devices.
5. Effectiveness: Does this system improve accessibility to safety practices? Considering the performance of the system by addressing required safety contents in a specific time and place through the visual interaction.
6. Time: How much time you must spend to make safety planning on this system? Considering spending time in comparison of WPT system and conventional method to develop a safety planning based on participants’ experience.
7. Upgradability: Can the system be improved for changes in the market? Focusing on the ability to update and synchronize TSFs information from the changing of supply market and project progress.

**Figure 14:**
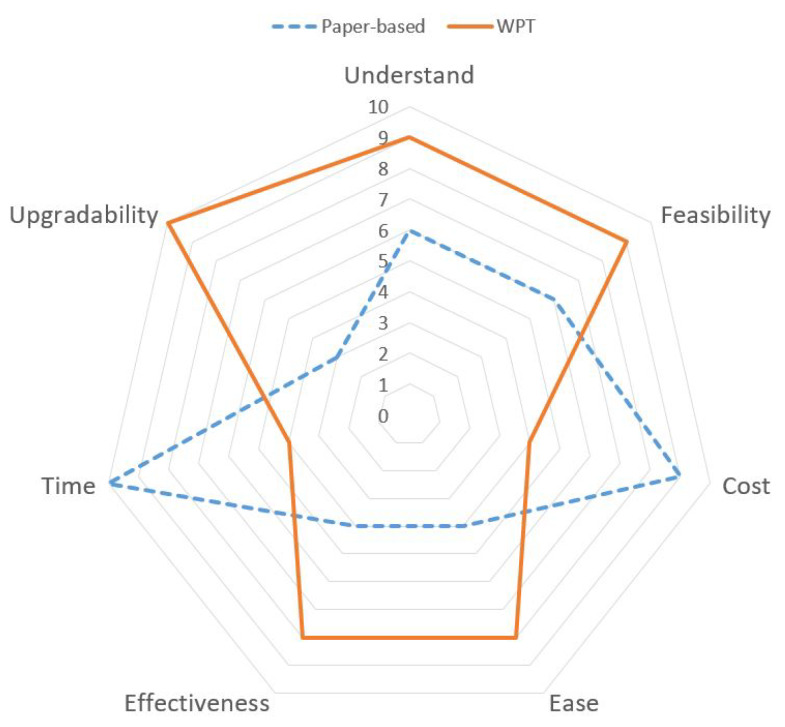
WPT system evaluation results.

The evaluation interview ranged from 10 to 15 min per people, and grade on the 10-point scale of satisfaction. Figure 14 shows criteria evaluation results for the comparison of using conventional paper-based approach and WPT system approach. Receiving positive feedback from participants, the qualitative comparison results proved that WPT is not only interacting friendly but also time-effective and cost-effective. The interviewer agreed that the traditional paper-based approach is popular but inadequacy and difficult to adapt to smart construction transformation. The WPT system presented the great potentials for widespread safety information and its application in small construction sites. The proposed approach saves time and resources required for safety planning process. Thus, it is affordable to SMEs based on adaptive aspects of construction information synch speed and long-term investment benefits that essential for SMEs’ development.

## 8. Discussion

The popularization of BIM is an opportunity and also a challenge to its effective application for all construction participants including both large scale and SMEs. This study proves the scalability of BIM and the application of graphical algorithm for automatically allocating schedule-based TSFs in small or medium constructions. The intention of targeting construction SMEs in this research is to motivate them to apply the digital transformation in their everyday work. Multiple steps are performed to transfer schedule data thorough an understanding of Excel and Dynamo.The main function of WPT system is to automatically provide the prioritized TSF recommends for a specific time and workspace. When using this system, cost-saving can be achieved with the reduction of labor resources and deficiencies in safety management. It also saves time and reduces accidents because of the automation, convenience, and accuracy of the system (e.g., automatically recommend TSFs, quickly deliver construction information, and reduce defects and incidents in construction site) compared to the conventional method. The spreadsheet used for quantity take-offs of the TSFs can be exported from the BIM environment to the Excel format. When the category and quantity of required TSFs are identified, SMEs will have many options of choosing TSFs with proper materials, styles, and prices on the supply market. This offers efficient data for detailed quantity estimation of TSFs, it also plays a critical role in reducing time and labor’s workload in cost planning process.

Despite a lot of potential with the involvement of this Autodesk’s add-in, the integration outlined in this paper has some limitations. Firstly, there is a restriction in mapping safety information for a specific duration with model objects information that requires manual review when safety contents are distributed to these components. Some regulations are designed for general construction activities, and some others are formulated for a distinct activity period; therefore, safety regulations cannot be considered solely based on object information. Sometimes it must be divided by conducted time. It is necessary to define such rules clearly in the 4D-BIM for each planned activity, and then develop an ID system to match the related information accurately. In addition, it is also necessary to provide ideal and accurate schedule information to the 4D-BIM platform to update it with specific safety tasks and conditions. The assessment method proposed in this study focused on the Fatal Four; the remaining accident factors that account for 40 percent of all construction fatalities would be explored in future studies. Arguably, the accuracy of the proposed concept depends on the number of fatality cases evaluated in calculating the accident rates by severity. Further research would be necessary to collect more data on fatalities and analyze the rate of fatalities in construction SMEs.

## 9. Conclusions

The paper contributes to the investigation of pros and cons of construction SMEs in implementing safety requirements. The research has identified the importance of automated workspace planning for TSFs to SMEs application. Our paper proposed a WPT system for automatically providing the prioritized TSF recommendations for the schedule-based spatial and temporal. By collecting Fatal Four data from OSHA website, categorizing them by construction sequences, investigating OSHA regulations for using activity-based TSFs and getting the advantage of BIM and VLP, a residential building case study was developed to prove the application of WPT system. Consequently, this paper proposed workplace planning focusing on the operational requirements and constraints of construction SMEs, which contribute to facilitate the safety planning process, enhance safety management, reduce manager workload, simplify worker involvement, and increase the information exchange between participants.

The proposed framework comprises two main modules: (1) hazard investigation, which identifies serious hazards related to activities and their corresponding safety measures; (2) hazard elimination, which provides visual safety planning with semi-automatic schedule-linked temporary safety measures. The WPT approach has the advantage of saving time and labor resources required by construction SMEs to implement the safety requirements. This results in considerable time and cost savings apart from ensuring safe working conditions for workers. To comprehensively evaluate the effectiveness of the proposed system, future work would conduct an objective comparison between the conventional and WPT approaches with the full-scale WPT systems.

On the other hand, the FFA-based computer vision application to remotely monitor and calculate the severity of accidents should be experimentally validated. Additionally, it would also be worthwhile to consider the effect of utilizing artificial intelligence (AI) to automatically complete the FFA process and recommend accurate risk management strategies. We also plan to integrate the system with AR technologies to deliver a real-like experience to users in onsite construction. The proposed approach will be validated further by applying it to real construction sites in Vietnam and South Korea to compare the practical applicability of this system in a developing and developed country.

## Figures and Tables

**Table 1:** Studies on the use of building information modeling (BIM) for safety planning.

Ref.	Research Contents	Technology	Hazard	Focus on	Safety Prevention
[12]	Safety planning process that addresses activity safety from accident data and activity risk factors.Safety analysis was performed by assigning scores to construction occupation type, accident type, and sources of injury.	4D-BIM	SA	Work zone	N/A.
[15]	Fall hazard detection and prevention by automated safety rule-checking algorithms for BIM model.	4D BIM	SA	Fall hazard	Guardrail.
[20]	Address potential safety issues of scaffolding. Automatically identify hazards related to each activity.	BIM	A	Scaffolding work	Scaffolding.
[21]	Automated analysis building geometry and safety plans for scaffolding structures considering their costs and duration.	BIM	A	Scaffolding work	Scaffolding, guardrail.
[38]	Identifying fall hazards and extracting relevant safety rules related to excavation.	BIM, Point cloud	A	Excavation and trenching	N/A.
[27]	Establishing automated workspace visualization using remote sensing and workspace modeling technologies for construction safety planning.	BIM, GPS	A	Workspace conflict	N/A.
[39]	Organize, store and re-use construction safety knowledge by ontology. Automated safety planning for Job Hazard Analysis (JHA) using BIM.	BIM, Ontology	A	JHA	Guardrail.
[40]	BIM-based framework for multi-objective and dynamic temporary construction site layout design considering cost and adjacency factors.	BIM	N/A	Site layout design	Temporary facility.

Note: (A) Automatic Identification, (SA) Semi-Automatic Identification, (N/A) Not applicable.

**Table 2:** Example of data obtained from FFA database.

Safety Contents	Example
Accident Date	20180105
Accident description	Employee Falls From Roof And Is Killed.
Accident subject	Open Skylight.
Fatal Four Classification	Falls.
Work activity	Roof Coatings.
Safety regulation	OSHA: 29 CFR 1910.23, 29 CFR 1926.500-503.
TSF Recommendation	Temporary construction guardrails or cover, Safety Harnesses.

**Table 3:** Safety data sourcing.

Fatal Four	Hazard Scenarios	Related TSFs
Falls	Unprotected Sides, Wall Openings, Floor Holes	Guard railing.
	Improper Scaffold Construction	Scaffold.
	Misuse of Portable Ladders	Ladders.
Struck by	Vehicles	Safety Labels.
	Falling/Flying Objects	Safety net.
	Constructing Masonry Walls	Limited zone.
Electrocutions	Contact with Power Lines	Safety Labels, Extinguisher.
	Path to Ground Missing or Discontinuous	Ground-fault Protection.
	Equipment Not Used in Manner Prescribed	Safety Labels.
	Improper Use of Extension and Flexible Cord	Safety Labels, Extinguisher.
Caught-in between	No Protective System	Stripping.
	Unsafe Placement	Marking tape.
	Unsafe Access/Egress	Marking tape.

**Table 4:** OSHA safety regulation for masonry works in the unprotected side.

Work Level	Wall Height	Protection	Method
		Guardrail	Limited Zone
H=0 m	H>2.4 m	No	Yes
H>2 m	H<2.4 m	Yes	No
	H>2.4 m Solid	No	Yes
	H>2.4 m Opening	Yes	Yes
